# The social dynamics of consent and refusal in HIV surveillance in rural South Africa

**DOI:** 10.1016/j.socscimed.2012.11.015

**Published:** 2013-01

**Authors:** Lindsey Reynolds, Thomas Cousins, Marie-Louise Newell, John Imrie

**Affiliations:** a Department of Anthropology, Johns Hopkins University, 3400 N. Charles St., 404 Macauley Hall, Baltimore, MD 21218, United States; b Africa Centre for Health and Population Studies, University of KwaZulu-Natal, Somkhele, South Africa; c UCL Institute of Child Health, University College London, London, UK; d UCL Centre for Sexual Health and HIV Research, University College London, London, UK

**Keywords:** South Africa, Ethics, Surveillance research, Obligation and reciprocity, Kinship, Local relations, HIV testing, Participation

## Abstract

In the context of low rates of participation in a prospective, population-based HIV surveillance programme, researchers at a surveillance site in rural KwaZulu-Natal, South Africa, conducted an operational study from January 2009 to February 2010, with the aim of improving participation rates, particularly in the provision of dried blood spots for the surveillance. Findings suggest, firstly, that consent to participation in the HIV surveillance is informed by the dynamics of relationality in the HIV surveillance “consent encounter.”

Secondly, it emerged that both fieldworkers and participants found it difficult to differentiate between HIV *surveillance* and HIV *testing* in the surveillance procedure, and tended to understand and explain giving blood under the aegis of the surveillance as an HIV test. The conflation of surveillance and testing, we argue, is not merely a semantic confusion, but reveals an important tension inherent to global health research between individual risks and benefits and collective good, or between private morality and public good. Because of these structural tensions, we suggest, the HIV surveillance consent encounter activates multiple gift economies in the collection of blood samples. Thinking beyond the complex ethical dimensions provoked by new forms of long-term surveillance and health research, we therefore suggest that deepening relations between scientists, fieldworkers, and study participants in locality deserve more careful methodological consideration and descriptive attention.

## Highlights

► Consent and refusal to HIV surveillance take place in the context of everyday social relations and local exchange economies. ► Local notions of gift and relatedness and rights and obligations frame exchanges of blood for knowledge in HIV surveillance. ► Participants struggled to differentiate HIV surveillance from testing, thereby blurring concepts of risk and benefit. ► Surveillance programs should engage with local constructions of risk, benefit and relatedness in research design.

## Introduction

The paper explores the dynamics of consent for the collection of dried blood spots (DBS) in the context of an ongoing prospective population-based HIV surveillance programme in a rural district in northern KwaZulu-Natal, South Africa. Based on data from the HIV surveillance programme, researchers have documented both high HIV prevalence (24.1 percent of the total population surveyed in 2010 were HIV-positive) and continued high rates of HIV incidence (the infection rate per 100 person-years was 3.8 in women aged 15–49 years [95% CI, 3.2–4.6] and 2.3 in men aged 15–54 years [95% CI, 1.8–3.1] in 2008 (Bärnighausen et al., 2008). In this context, though the linking of HIV surveillance data to demographic data on household dynamics, socio-economic status, population mobility, and relationships have made it possible to adjust for selection effects in HIV prevalence and incidence estimation (Bärnighausen et al., 2008), declining consent rates for the collection of dried blood spots in the HIV surveillance raised concerns about the strength of HIV incidence estimates drawn from the surveillance data. As one of the few sites in South Africa conducting prospective population-based HIV surveillance, both the incidence and prevalence data generated through the surveillance programme are important for research, policy and programming in order to estimate the current and future needs for HIV prevention, treatment and care.

The material presented here is drawn from an operational review undertaken at the Africa Centre for Health and Population Studies in 2009 in order to better understand the factors underlying declining consent rates, particularly the collection of DBS for HIV testing, and to identify strategies to increase participation. This paper reflects on the lessons learned in the operational review process, and explores broader questions regarding the dynamics of research and reciprocity in the context of HIV research.

The Africa Centre for Health and Population Studies (hereafter “the Africa Centre”) was established in 1998 as part of an international collaborative research programme. Since the start of the centre's operations, the surrounding population has participated in ongoing demographic and health surveillance as well as numerous social science and health research projects and clinical trials. While the demographic surveillance has generally had high rates of participation, participation in the HIV surveillance has been an ongoing challenge. The extraction and circulation of blood in the HIV surveillance, symbolically mediated by the DBS, appears to cast social relations in a more difficult and complex light. Rather than a diagnosis of potentially faulty operations or field relations, the research explored the operational processes and institutional relations between respondents and fieldworkers, managers and scientists, formal institutions and social conventions that have structured the ways in which the HIV surveillance has been experienced and thus consented to or refused. The factors that shaped rates of consent for HIV surveillance can best be understood, we suggest, by examining the experiences that participants and fieldworkers bring to the “consent encounter.”

Our findings suggest, firstly, that consent and refusal to participate in HIV surveillance take place in the context of densely layered social relations. The collection of the DBS embodied a form of knowledge that brought to the fore questions of sociality, secrecy, confidentiality and trust entangled within complex forms of relatedness in this locality. To understand these tensions, we suggest, consent and refusal to HIV surveillance should be framed in the context of local idioms of relatedness that in South Africa have made understanding the HIV epidemic and controlling it particularly difficult. Secondly, it emerged that both fieldworkers and participants found it difficult to differentiate between HIV *surveillance* and HIV *testing* in the surveillance procedure. For many respondents, “giving blood” under the aegis of the surveillance was understood simply as an HIV test, with all its accompanying significations: the social technology of counselling, delivery of results, public shame, personal fear and the embodied experiences of illness and treatment. The conflation of surveillance and testing, we argue, reveals not only the distinct social and epistemological significances of the ‘gift of blood,’ but also the tensions between individual risks and benefits and collective or public goods.

The long history of medical research in Africa has raised many questions about the role of health research organizations in colonial government and thus about the need for more just and ethical research practices in post-colonial Africa (Crozier, 2007; Feierman & Janzen, 1992; Packard, 1989; Vaughan, 1991). A recent ethnographic literature on demographic surveillance and health research has taken up the way in which medical ethics have been reformulated in response to these dilemmas by developing stronger protections for individuals and a clearer population concern with justice in regard to wider inequities in health and wealth (e.g., Fairhead, Leach, & Small, 2005; Farmer, 2002; Geissler, Kelly, Imoukhuede, & Pool, 2008; Gikonyo, Bejon, Marsh, & Molyneux, 2008; Molyneux & Geissler, 2008; Stewart, Keusch, & Kleinman, 2010). In a 2008 Social Science and Medicine special issue on “trial communities” in Africa, for example, scholars explored the tension between formal ethical frameworks, which are often bureaucratic and generalizing, and more localized systems premised on what Geissler et al. (2008) term “ethical relations,” characterized by their fluidity, contingency and intimacy. Many of these studies conclude in a similar vein by advocating not simply for changes in study design, but for major shifts in the “very real political economy of the global medical research industry” (Fairhead, Leach, & Small, 2006, p. 1118).

As these articles suggest, hierarchised notions of local/global actors or domains obscure the grounded practices that give such social technologies their character (Ferguson, 2006; Geissler et al., 2008; Latour, 1988; Stewart & Sewankambo, 2010). In addition, they reveal how liminal figures, such as field workers, are often forced to mediate tensions between the norms and standards of global health research and local life worlds. While such liminal figures deserve much greater ethnographic attention for their role in giving voice to local ethical concerns, we argue here that the ambivalent configurations of kinship, obligation and trust produced in such “trial communities” (Molyneux & Geissler, 2008) crucially shape the conduct of HIV surveillance and thus the production of knowledge emerging from such techno-social contexts. Understanding the ambivalent socialities generated in the HIV surveillance encounter, and the ways they shape concepts of risk and benefit, is vital to improving participation in long-term surveillance and global health research more broadly.

## Methods

The operational review took place from January 2009 to February 2010 as an internal exercise intended to understand and address declining consent rates, and thus no prior ethics approval was sought. However, this manuscript was reviewed and its publication approved by the University of KwaZulu-Natal Biomedical Research Ethics Committee (BREC) on the grounds that 1) the work began as an operational review; 2) publication was only considered by the authors when the results appeared to be of broader value and interest; and 3) BREC approval was expressly sought for publication. All identifiers have been removed to ensure the anonymity of all respondents and participants.

To examine the conditions that frame the lives of individuals within the context of a population surveillance programme, we used an ethnographic approach that elucidates both the dynamism of social relations and the intricacies of everyday experience. Additionally, we used a ‘process approach’ within the organisation, through which findings were folded into programme design and staff training. The first phase of the review focused on understanding how the surveillance programme functioned and how its aims and procedures were understood. We conducted interviews with principal investigators, researchers and field supervisors; reviewed training manuals, recruitment scripts, consent forms, operational guidelines and publications related to the HIV surveillance programme; and conducted field visits (*n* = 25) with data collectors, counsellors and community liaison officers to observe encounters between data collectors and respondents. These visits formed the basis for a series of twelve focus group discussions with HIV surveillance field staff.

The next phase aimed to explore respondents' experiences and understandings of the surveillance programme. We began by conducting a series of discussions with members of the centre's community advisory board, a group of approximately 25 members representing key constituencies within the demographic surveillance area (DSA). Board members then referred us to other key informants within the community, with whom we conducted nine in-depth interviews. In addition, we conducted semi-structured interviews with 25 individuals in areas with both high and low rates of consent for the DBS.

## Contextualising the surveillance encounter

The social setting in which surveillance is conducted is important, as well as the techniques and technologies assembled, in order to locate the labour of the fieldworker in the surveillance consent encounter. The Africa Centre was conceived in 1997 as a collaboration between the Universities of Natal and Durban Westville (now combined in the University of KwaZulu-Natal) and the South African Medical Research Council, with funding from the Wellcome Trust, the largest health research foundation in the UK. Around two hundred kilometres north of Durban, the district where the Africa Centre is based is one of the poorest in KwaZulu-Natal (Case & Ardington, 2006). The Africa Centre defined a bounded area of 438 square kilometres near to the market town of Mtubatuba in the Umkhanyakude district of KwaZulu-Natal as the Demographic Surveillance Area (DSA). Though the area is physically hemmed in by both natural and man-made boundaries (rivers to the north and south, a game park to the west and a major national freeway to the east), the population is fluid, with high rates of mobility and migrant labour. The designated area includes land under the Zulu tribal authority that was formerly classified as a homeland under the Apartheid-era Bantu Authorities Act of 1951 (Crankshaw, 1993), as well as an urban township, formerly designated for ‘African’ residents, under municipal authority. Though the area is often defined as ‘rural’ South Africa, there is a large variation in population densities—from 20 to 3000 people per square kilometre (Tanser et al., 2008). Similarly, infrastructure development across the area is heterogeneous, ranging from fully serviced ‘modern’ houses to isolated homesteads without water, electricity or sanitation.” Livelihoods in the area are deeply structured by the political and economic history of the former KwaZulu Bantustan, of which it formed a part, and are characterised by high levels of unemployment and reliance of government welfare payments. The population of approximately 90,000 (resident and non-resident) individuals living in 11,000 households is almost exclusively isiZulu-speaking. Information about these subjects, including mortality, fertility, and migration, is stored longitudinally in a single database (Tanser et al., 2008).

Since 2003, a population-based HIV surveillance programme has been nested within the demographic surveillance. All data collected in the HIV surveillance is spatialised and can be linked anonymously to longitudinal data collected in the household surveillance. Like many other demographic and health surveillance sites in Africa, the Africa Centre has also over the years served as the site for a variety of clinical trials and other health research projects. At the time of this research, the Africa Centre was also receiving PEPFAR funding to support the Department of Health in implementing the Hlabisa HIV Treatment and Care Programme within the research area. The program had enrolled over 11,000 individuals, making it the largest rural treatment program in the country.

Through 2009, each HIV surveillance round consisted of 40 “week-blocks” during which eligible participants were asked to provide a DBS sample that was tested for HIV as part of an anonymous linked surveillance programme, as well as to answer questions related to their health and sexual behaviour. Between 2003 and 2006, all women aged 15–49 years and men aged 15–54 resident in the surveillance area were eligible to participate in the HIV surveillance programme. In 2007, eligibility was extended to cover all residents aged 15 years and older. The HIV surveillance completed its 6th round in 2009, during which fieldworkers sought to collect a blood sample from every eligible resident adult individual registered in the surveillance database, a total of 34,278 individuals.

In round 6, “participation” in the context of HIV surveillance entailed several opportunities to consent or refuse. Firstly, residents could refuse the surveillance encounter entirely through either outright refusal or passive avoidance. They could also consent to specific parts of the surveillance (health and/or sexual behaviour questions) while refusing to provide a DBS, or could provide a DBS and not consent to receive their HIV test results. To describe the many choices provided to participants, the 2009 HIV surveillance consent form consisted of four pages of written information. The form first explained the purpose of the surveillance: “to help us learn more about HIV as well as about prevention, and through this provide information on how to improve the services that are available to you and your community…." The explanation was then followed by ten separate requests for consent. These included: “to have a few drops of blood taken through a finger prick which will be tested for HIV” (the DBS), to have the blood sample stored, to perform ARV screening and resistance testing on the blood sample, to answer questions about health, to answer questions about sexual behaviour, and to be contacted by researchers regarding other studies. Further, respondents could opt to receive the results of the HIV test performed on the DBS at their home, to collect the results at the counselling centre in the nearby town; to be referred for a separate rapid HIV test; or not to receive HIV test results at all.

For the purpose of the operational review, we worked with “consent rate” defined as the proportion of contacted individuals who consent to give a DBS over the proportion of people contacted by fieldworkers (not all eligible residents). Using this definition, participation in the DBS collection was 58 percent at its highest and 32 percent at its lowest over the six years from 2003 to 2009. While it is clear that consent rates declined from the first round to the sixth round, it is important to note that by the end of 2009 nearly two thirds of the eligible population had consented to provision of a DBS at least once since the beginning of the HIV surveillance programme. This may suggest that a significant part of the problem with declining consent is linked to the prospective nature of the study—to the fact that individuals have been asked to give their blood each year, irrespective of their HIV status. Thus it is important to make sense of both the relatively low annual participation rate and the higher cumulative participation, a point we draw out below.

In 2009, we spent time with HIV surveillance fieldworkers to observe their daily labours and their interactions with research participants. Surveillance fieldworkers begin and end every work day at the research centre. The centre sits in the middle of the DSA in a prominent and easily recognisable building encircled by a series of smaller buildings and parking lots. The entire complex is walled off from the surrounding area by a security fence. Outside the gate a crowd of minibus taxis drop off and collect workers at designated times each day. Upon arrival for work each day, staff members must swipe identification badges to open a large gate, watched over by private security guards. There they meet up with their teams, each consisting of four male/female pairs, and receive their instructions, maps and forms for the day's visits from their supervisors. They then head out for the day in a fleet of white Land Rovers, emblazoned with the centre's logo.

Over the course of three months in early 2009, we accompanied the teams as they drove to designated areas within the DSA, and then walked with fieldworker pairs as they travelled over the hills and along paths from one rural homestead to the next. The surveillance protocol dictated that when fieldworkers arrived at a homestead, they should announce themselves, pay respects to elders in the house, explain their intentions and invite eligible adults to participate. It was at this moment that the formal consent process was interpreted by the fieldworker, and where they were required to translate the technical language of bioscience into an experientially meaningful picture of participation.

In most encounters, the fieldworker would briefly mention the many elements of the consent form, often condensing and adapting them as they interpreted the response of the person before them. Participants were then asked to sign their name and the fieldworker signed below. Despite the difficult subject matter, we generally found this moment to be held politely by fieldworkers and respondents, and refusals to be soft and indirect. In addition, as we explore below, the consent encounter produced for fieldworkers an acute sense of expectation that the Africa Centre reciprocate in some manner for the intrusion into delicate personal matters.

A visit could be short if none of the eligible individuals were present or up to an hour if several eligible individuals were present and gave consent to the DBS collection and health and sexual behaviour questionnaires. Once consent was given and the DBS procedure explained, the fieldworker would don surgical gloves, dispose of the old needle in the medical waste bucket, and ready the finger-lancet with a new needle. The fieldworker would then rub the respondent's finger with alcohol and prepare the contact sheet by recording details and attaching barcodes. Barcodes on blood samples and surveys ensured confidentiality. After pricking the finger, the fieldworker would collect five drops of blood on the sheet in neat circles; seal it in a clear plastic layer and file it. The participant was then given a cotton swab to clean the prick, and the interview would continue. The research protocol thus coordinates a series of gestures and exchanges that are critical to establishing relations between fieldworker and participant.

At the end of each day, fieldworkers returned all samples and surveys. Correctly completed surveys were sent for data capture and blood samples were driven to the laboratory 2 hours away. Then HIV test results were emailed to the data centre where they were downloaded onto Palm Pilots for counsellors to deliver to participants' homes. A separate team would return to deliver results to individuals who had consented to receive their results. Fieldworkers must cover the 40 week-blocks annually by traversing the DSA every weekday, no matter the temperature or the rain. The physical demands of data collection are an added stress for fieldworkers who mediate the social relations produced by the surveillance encounter.

## The gift of blood

The central concern emerging from focus groups, field visits and participant interviews was the confusion between HIV surveillance and HIV testing. The conflation of these two concepts occurred across many levels of the surveillance programme. This distinction was frequently obscured in the consent forms and other materials offered by the centre to participants. For example, in the 2009 consent form, the benefits to study participation were described exclusively as benefits related to knowing one's own HIV status:

People who know that they are HIV-negative will have greater reason to take precautions and remain negative. People who know that they are HIV-infected can benefit from counselling and support and pay greater attention to healthy living, which will increase their years of health. Anybody who is HIV positive can be referred by an Africa Centre counsellor to a local health facility for further clinical examinations if desired… People who know that they are HIV-infected can also prevent transmission of HIV to their sexual partners and to others.

In their work, fieldworkers were generally not able to make clear the distinction between the collection of blood samples for surveillance and the opportunities to test oneself for HIV, between the research goals and the services offered. Similarly, interviews with respondents and observations of the consent process suggested that though respondents had a general understanding of the technology of surveillance, most tended to conflate their refusal to give a DBS with reasons why they did not need or want to test for HIV. As one fieldworker put it, people refused because they were not ready to know their HIV status, because they already knew their status, because they doubted the reliability of the test or because they preferred to seek care from their own doctors. A 35-year-old man who had refused the DBS told us, for example, “When they came to us for blood, we had just tested with the counsellors at the clinic, so I told them that they can just go to the counsellors and check my file. So I did not give them my blood.”

None of our respondents felt able to give a DBS and not have an HIV test from which they would receive their results. Individuals who tested for HIV recently, who tested in a previous round or who were not prepared to test at the moment the fieldworkers arrived would refuse to provide blood in a given round. It is in this context that the distinction between testing and surveillance (or between personal benefit and collective good) takes on its valence and the question of the appropriate circulation of benefits for study participation comes into focus.

Part of the confusion regarding the purpose of, risks involved in and benefits derived from the collection of blood in the context of HIV surveillance may stem from the difficult history of the research centre's orientation to the question of services and benefits that accrue to the study population. Since the start of the programme, various forms of HIV testing services have been offered to participants in concert with the surveillance. At the start of the HIV surveillance in 2003, giving each individual the opportunity to know their HIV status in return for the provision of a blood sample for research purposes was considered a fair exchange of data for services in the context of the limited availability of HIV testing and treatment when the HIV surveillance began and it continued to be emphasised for the first six rounds of surveillance.

In each round, small changes have been made to the programme's operational design in response to concerns raised by fieldworkers, the Community Advisory Board and internal evaluation reports. For example, HIV test results were initially available to participants at 19 community centres around the DSA staffed by trained counsellors who lived in each locality for the duration of the surveillance round (Weltz et al., 2007). In 2007 rapid HIV testing was also made available during home visits. In response to perceived concerns around confidentiality, a home delivery system was then established in 2008 for linked, anonymous voluntary HIV testing with pre- and post-test counselling using confidential pin numbers and handheld devices for result communication (Bärnighausen et al., 2008). By 2009, however, as testing services became more widely available in the area, researchers began to speak of the benefits related to testing in different terms. Each change has complicated participants' perceptions of the HIV surveillance and has added a layer of operational complexity to the institutional history of the research centre within the social and political landscape of the DSA.

Because of these structural tensions, we suggest, the HIV surveillance consent encounter activates multiple gift economies (Schrift, 1997) in the collection of blood samples. In the circulation of blood, results and benefits, obligation and debt are invoked by both fieldworkers and respondents. Generally, the conduct of public health research is premised on the notion of voluntary participation and is permitted on the grounds of a greater good that it is imagined will accrue from the production of knowledge flowing from the collection of data. Similarly, voluntary participation in the HIV surveillance is premised in scientific discourse on an assumption that collective benefits derived from demographic and epidemiological research will accrue to “communities” located in the DSA. However, the many overlapping claims to community that emerge through the lens of both household and individual surveillance programmes and other research projects signal the difficulty of delimiting the HIV surveillance to specific communities in relation to particular benefits that may accrue to them.

In its research practices, the organisation has continually emphasized the two poles of “individual” and “community” benefits. For instance the *Operational and Methodological Procedures* states:

Since its inception, the Africa Centre decided to become embedded in the community in which it was placed. When conducting research on human beings it is both a moral obligation and a practical necessity to set up and maintain good relationships with the population one serves. Ideally, individuals and the community should feel themselves participants in the activities of the Centre…(Muhwava et al., 2008, p. 33).

Despite the scientific rhetoric on collective benefits, however, the basis upon which to encourage participation in the HIV surveillance has been the sense of service rendered to an individual in a direct, material way: ‘you are better off for knowing your status.’ The possibility of the gift thus establishes a tension expressed by managers, fieldworkers and participants, between modes of research and service provision, between the research goals of HIV surveillance and the potential benefits of HIV testing.

It was within the moment of the field encounter that the difficulty of asking for blood became a question of the gift and the difficulty of exchange. The intention of a fair exchange of blood for benefits is not easily reconciled for fieldworkers or participants. The logic of HIV surveillance has been cast as a form of gift, but the gift, the “benefit” of knowledge of one's HIV status in this instance, is closer to the sense of poisonous gift that Mauss outlined in his early essay (Mauss, [1924] 1990).

In our case, the blood samples enter into circulation along with concerns about the dangerous knowledge the blood embodies, and about the confidential handling of this knowledge as it is turned into anonymous data, while its substantiality provokes concerns about improper use by those with ill intent. It was clear that all respondents understood that any handling of blood implicated HIV in some way, but that the stakes entailed in the circulation of blood have made direct reference to it, and any “anonymous” donation of it, extremely difficult.

You know, we have now this general suspicion about blood. We’re talking just about a few drops, not donating a lot. But we are scared of blood. We know that this virus thrives in blood. So, are we safe even during the time of exchanging those few drops?" (Ward councillor)

Further, part of the difficulty people faced in specifying what is meant by “giving blood” was related, we suggest, to their discomfort around the question of their own HIV status. While resistance to HIV testing is continually shifting in South Africa (and many more people test now than before national provision of ARVs), many of the same factors that inhibit uptake of HIV testing services generally remain pertinent to the DSA population. Nearly all respondents spoke vividly about the fear of finding out one's HIV status. Of particular interest were the twin elements of fear associated with knowledge of one's status: first, one's own sense of wellbeing, in the context of an HIV positive diagnosis, and second, a more socially embedded fear of others finding out one's status. Even the programme's own consent protocol highlighted these two elements of concern in the enumeration of risks associated with study participation:

There are, unfortunately, potential risks to having a blood test for HIV and knowing your HIV status: People who learn that they are HIV-infected may suffer from mental stress and depression as a result. They may also be stigmatized by their family and community and may be discriminated against – although this is illegal in South Africa.

For many respondents, coming into knowledge of one's HIV status signals the potential moment of one's decline and slide into death. As one 45-year-old female participant explained, “I will just get so depressed if I am positive, I’ll just get sick, lose weight and die.” Further, fears around the implications of others learning one's status were central to concerns about providing blood in the HIV surveillance.

It is difficult, not easy, to know about your status. For some people, it is not a problem, they like to know. But some people are scared to come because they think now the whole world is going to know that in that house it is finished. Valiwe. It's closed. It's done (Female, in-depth interview).

Because of the risks associated with the provision of blood, many respondents gave expression to one of the central tensions articulated by HIV surveillance staff: the difficulty of participating without obvious personal gain. Many expressed this in terms of a critique of the research centre generally – “what have they done for us?” – and always in relation to what services other community organisations offered. When asked how she understood the consent rate, one fieldworker explained, “The low consent rate is because we do not give anything to the community. Every day we are coming and asking one and the same question and we’re taking the blood samples, but we do not give anything to the community.” A research participant articulated the challenge particularly clearly: "The community is interested in service, not research." These concerns have amplified over time as community members experience more and more research: "The community is exhausted now by all the studies, there are too many cars visiting the yard."

In every case, the discussion of benefits was expressed in relation to forms of care and assistance related to HIV. Thus, if the collection of a blood sample and administration of sexual behaviour questions were intended to bring health benefits to this community, then the research centre would have a responsibility to respond to the needs of those participants who were directly affected by HIV – either by a death in the family or a more everyday challenge, such as a lack of transportation to the clinic or insufficient food.

More broadly, participants tended to understand "research" as something akin to intervention, and indeed the use of the isiZulu word *ucwaningo*, used for both the “research” and “surveillance”, but also meaning "search and sift out the details of a matter; pick up small bits; ostracize” (Doke, Malcolm, Sikakana, & Vilakazi, 1990), across these settings points to the slippage and carriage of meaning here. This confusion could be construed as an instance of the 'therapeutic misconception' (Appelbaum et al., 1982), whereby research participants mistakenly imagine that the agendas of those involved in medical research are oriented toward their best interests, as would be the case in a clinical encounter. Stewart and Sewankambo (2010) argue that this conflation of health research with expectations of direct benefits cannot be seen as a simple 'misconception,' but rather is a reflection of “fundamentally different expectations for health research” (p.175).

In this context, the conflation of HIV surveillance and testing is not merely a procedural confusion. Rather, it signals a structural tension inherent to global health research, and offers us a lens through which to better understand social value. Through the language of participation, the informed consent process collapses individual risk and benefit and collective or public good. In the context of the HIV surveillance, the declining consent rate suggests a problematic formulation of reciprocity at work within the technology of the HIV surveillance, made manifest in the field encounter. Experientially, much more is at stake than a simple cost-benefit analysis of personal risk or collective benefit that has made it difficult for the fieldworker to explain and for the respondent to absorb the distinction between surveillance and testing, or between research and service. The HIV test is constructed as a technology of the individual; the HIV surveillance as a technique of population (cf Foucault, 1982). Together they require the voluntary participation of a particular kind of person who benefits from inclusion in the polis of public health. As Copeman et al describe, the capacity for blood to mobilize varying tropes of relatedness, inclusion, or citizenship is highly sensitive to local constructions of politics and technological solutions to questions of blood supply (2009).

The different consent rates between demographic and HIV surveillance confirm that the forms of relatedness at stake in the circulation of blood and knowledge of its status are entirely different from those entailed in knowledge of the “household.” Demographic surveillance requires the participation of only one key informant per household while HIV surveillance requires consent from every individual participant. Individual participation and individualised benefits cut across (and draw on) possibilities of personhood that invoke socially embedded forms of relationship. When fieldworkers are not able to explain carefully the collective benefits that accrue to large areas and groups of people because of the various research projects of the centre, the potential for resentment is compounded. All the associated expressions of confusion stem from the fact that socially and experientially, fieldworkers found it difficult to ask for the donation of a DBS sample without being able to offer something in return. This finding suggests that the public good promised by participation in HIV surveillance does not carry sufficient return or evidence in the experience of those being asked to participate. It may also suggest that the collective in whose name the research is conducted does not adhere sufficiently or carry enough social force in that moment of the exchange. It is thus possible to refer to (multiple) gift economies that operate in the shaping of everyday relationships between people and institutions in the social landscape of the DSA.

## Intimacy, secrecy, trust

As many scholars of HIV in southern Africa have noted, the politicisation of sexuality in the post-Apartheid era and the controversial denialism of former president Thabo Mbeki have contributed to acute anxieties about testing for and living with HIV (Jewkes, 2006; Posel, Kahn, & Walker, 2007; Young et al., 2010). Aspirations of a better life after the degradations of Apartheid have been confronted by increasing inequality and the social effects of AIDS-related mortality (Posel, 2005; Seekings & Nattrass, 2005). In response to moral crises presented by HIV to social networks and livelihoods, public health interventions have sought to responsibilise and conscientize youth, mostly in urban centres (Colvin, Robins, & Leavens, 2010; Robins, 2006; Robins, 2008), although the problem of how to understand sexual networks across urban and rural sites, and the difficulty of testing for HIV has recently gathered attention (Steinberg, 2008; Thornton, 2008).

The densely layered social networks within and across the boundaries of the DSA make HIV testing a public experience and being seen to test thus places those networks in question. In this context, making explicit the purposes of the surveillance entails an interpretive moment for participants who must consider the particular social terrain of the locality in which the surveillance programme operates. Blood relations remain an important mode of political life in post-Apartheid KwaZulu-Natal. Unlike in other parts of Africa, people in this area live in scattered homesteads rather than in clearly bounded villages, making definitions of ‘community’ a complex task. Migrant labour remains a vital mode of livelihood for this population, which practices very little subsistence agriculture, and state welfare assistance has become an increasingly important feature in domestic economies. The DSA itself falls in an area with multiple overlapping forms of governance, reflecting the complex political history of South Africa and of the former KwaZulu Bantustan. Additionally, social institutions that draw people into diverse modes of belonging, such as the many Pentecostal denominations, mutual aid and burial societies, employers, and family networks, also cross the boundaries of the DSA.

In this context, limiting the circulation of the poisonous knowledge of one's HIV status was an important concern for study participants. Thus, a central issue brought up by nearly all respondents was the relationship between fieldworker and research participant. Our findings suggest that forms of sociality that enliven local relationships are an important element in decision-making regarding anonymous HIV testing.

The work of Geissler et al. (2008) on relational ethics and material exchange involved in the giving of blood in the context of a malaria vaccine trial is an excellent example of a critical reconsideration of the kinds of relationships that emerge between study participants and researchers in long-term research engagements. Of particular interest to us is the way in which the symbolic associations of blood and its circulation carry the capacity to enliven or mobilise kin relations in the research activities and service provisioning of trial communities. While the possibility for positive invocations of kinship emerge in some settings from the intersections of discourses of blood and practices of inclusion, in others they can be more destructive (Erwin, Adams, & Le, 2009). Similarly, our data from the Africa Centre's DSA suggest an ambivalent invocation of kinship that places questions of trust, suspicion and secrecy in a complex light. A broader economy of secrecy and sexuality appears to condition the field encounter such that the circulation of blood in seemingly mysterious ways places kin and other relations in tension. Relatedness thus can also entail suspicion and secrecy, denial and doubt, or contradictory expectations and obligations.

Fieldworkers in both demographic and HIV surveillance teams are preferentially hired from within the DSA as one form of “community benefit.” However, hiring local staff raises a concern that greater familiarity between fieldworkers and residents may form an obstacle to donating blood samples to the HIV surveillance because of a perceived risk of disclosure of HIV status, the circulation of that dangerous knowledge and the possible negative consequences of public humiliation. The difficulties of asking for blood samples and for details of sexual behaviour cut to the heart of the problem of consent: these questions require a degree of familiarity and intimacy that the construction of professional relationships cannot contain. The potential for one's HIV status to be known, the intrusion into family spaces required, and the discomfort of talking about personal health concerns and blood to those more junior or of the opposite sex, have made the work of the fieldworkers especially complex. Interestingly, respondents often expressed divergent opinions as to whether it would be easier to give a DBS to a fieldworker who was familiar or to a fieldworker who was completely unknown. While some said that involvement in the research would be easier if the fieldworker were senior, trustworthy and known, most expressed that it would be better for the fieldworker to be a complete stranger, someone unknown in the area.

People around here would not want a boy from a Zulu household to know their status because he would tell others. That's a problem. Sometimes the fieldworker is someone you do not know, sometimes it is someone you know. A boy who grew up in front of me, I will just tell him off (Male, 40, in-depth interview).

Many fieldworkers confirmed this discomfort with asking relatives for blood: “It's difficult for them to consent because they are my relatives in this area” (Female fieldworker, 41, focus group discussion). Alternatively, however, in the same discussion group, one fieldworker pointed out that “sometimes people give their blood as a favour to the fieldworker – even if they’re on ARVs already” (female fieldworker, 34, focus group).

Fieldworkers who reside and have long histories of social ties within the area become mediators of diverse and sometimes contradictory interests – of the households of which they are members, of the communities from which they hail and of the research programme and its objectives. Further, given the high levels of unemployment, their status as trained and qualified professionals is not unproblematic for many people, making them potential objects of envy and distrust.

## A new surveillance framework

Because of the possibility that fear surrounding knowledge of one's HIV status contributed to the lower-than-desired consent rates, the need for a clear distinction between HIV testing and HIV surveillance research in the collection and analysis of blood became central to the HIV surveillance research agenda in 2009 and 2010. Re-articulating the exchange embedded in the surveillance encounter away from a sense of personalised knowledge and service to a more anonymous, aggregated collection of samples and community benefits was a key focus of changes to the surveillance programme in the 2010 round.

After an extensive ethical review process, a new protocol was rolled out in early 2010. The surveillance study name was changed from “Population-Based Testing and Counselling for HIV” to “Population-Based Biomeasures of Adult Household Members.” Another component of the project was added focusing on the collection of height, weight and blood pressure of participants, in addition to blood samples, which had first been collected in 2003/2004. In lieu of offering HIV testing services within the context of the surveillance programme, mobile testing units from the Department of Health Hlabisa HIV Treatment and Care programme would visit households after surveillance had been conducted to offer voluntary counselling and testing as a separate service. Further, in the new consent form and information sheet for HIV surveillance, nearly all of the language regarding HIV ‘testing’ was removed and replaced with the term ‘analysis’ (e.g. “The blood specimen will be sent to the laboratory for analysis”). Instead of listing the benefits of HIV testing, the new form focussed on the community-level benefits of research participation: “By participating in this study, you are helping the government to plan better services that will benefit you and your community eventually.”

In the new surveillance programme, the economy of information and consent regarding participation in the surveillance programme must be articulated purely in terms of “community” benefits that all partake of; surveillance thus becomes a public good, in which the various publics hereby imagined are inclusive of *isigodi*, tribal area, DSA, and the nation at large. It remains unclear how fieldworkers and respondents have been negotiating this new configuration of risks and benefits and re-imagining ideas of reciprocity in relation to the HIV surveillance programme.

## Conclusion

Our study of the dynamics of consent in the context of long-term prospective population-based HIV surveillance revealed two key findings. First, consent and refusal to participate in HIV surveillance should be understood in the context of everyday forms of sociality that bring together concerns such as institution and individual, power and inequality, science and tradition and research and intervention. Secondly, there was a confusion of HIV surveillance and testing in participants' and fieldworkers' experiences, which offers a lens through which to understand questions of social value in this locality. The conflation of surveillance and testing structured researchers', fieldworkers' and study participants' experiences of the tensions between individual participation and collective benefit. Though the imagined collective's members may or may not identify themselves as a coherent social group or political entity, their collective well-being is staked by the seemingly mysterious way in which the HIV epidemic travels, and the benefits of biomedical and demographic research that are claimed in the name of the collective good. In contrast, the personal crisis of an HIV positive status articulates with the lived importance of diverse modes of relationship that draw people together in complex associational forms in post-Apartheid South Africa. We thus need to incorporate into an understanding of “refusal to participate” a more complex picture of the tensions that are brought to bear on the consent encounter.

As new government welfare policies begin to shift local health care services in post-Apartheid South Africa and beyond, and new orientations to HIV and health research emerge worldwide, it is critical that research design remains alive to these shifting social realities. As other scholars have shown (e.g., Geissler & Molyneux, 2011; Kelly & Geissler, 2011; Lock & Nguyen, 2010), ethnographic attention to local life worlds is vital to the study of long-term global health research in such rapidly changing circumstances. Lastly, reflective practice processes that are participatory, build capacity, and clarify aims and methods, not only allow for more robust research but also remain open to the possibility of more equitable outcomes for health.
